# First Aid to the Injured and Sick

**Published:** 1902-03

**Authors:** 


### Books and Magazines.

First Aid to the Injured and Sick. By F. J. Warwick, B. A., M. B. Cantab., Associate of King's College, London; Surgeon-Captain, Volunteer Medical Staff Corps, London Companies, etc.; and A. C. Tunstall, M. D., F. R. C. S. Ed., Surgeon-Captain Commanding the East London Volunteer Brigade Bearer Company; Surgeon to the French Hospital and to the Children's Home Hospital, etc. 16mo volume of 232 pages and nearly 200 illustrations. Philadelphia and London: W. B. Saunders & Co., 1901. Cloth, $1.00 net.

The first part of this little volume is devoted to elementary anatomy and physiology. The bones, joints, soft parts, blood, respiration, digestion, etc., are all described in a readible and easily understood manner.

Part II deals with emergency bandaging, the treatment of hemorrhage, wounds, sprains, dislocations, fractures, burns, insensibility, fits, poisoning of various kinds, and the like.

The illustrations are excellent, and aid materially in making the text easily understood.

The book is of course written primarily for the use of laymen, but medical students, and even practitioners, will do well to have a copy on hand. Being printed on thin paper, it can easily be carried in a small pocket, notwithstanding its 232 pages. One dollar is a merely nominal price for such a book. R.

